# NUTRITIONAL NEEDS AND PREFERENCES AMONG AFRICAN AMERICAN CUSTODIAL GRANDMOTHERS: A QUALITATIVE STUDY

**DOI:** 10.1093/geroni/igz038.2343

**Published:** 2019-11-08

**Authors:** Kellie E Mayfield, Deborah Whitley, Susan J Kelley

**Affiliations:** Georgia State University, Atlanta, Georgia, United States

## Abstract

This presentation summarizes a qualitative analysis from focus groups with African American, urban dwelling grandmothers raising grandchildren in parent-absent households. Nutritional needs of custodial grandparents are an under explored area of research. Previous studies on custodial grandparents have acknowledged the physical, social, and familial burdens they endure as caregivers of their grandchildren. Limited financial support is a consistent concern. One manifestation of having scarce monetary resources is not being able to meet daily nutritional requirements. As a result, adverse health outcomes related to the onset of diet-related diseases (e.g., obesity, hypertension, diabetes) are too common, especially for custodial grandparents of color. The present study qualitatively explores grandmothers’ (N=9) experiences and ideas about food choices/options, decisions about when and where to purchase food, and the involvement of grandchildren in food-related practices and traditions. Grandparent participants were recruited from a community-based intervention, a program that provides health and social support services to grandparents raising grandchildren in Atlanta. Each of the focus groups consisted of 4-6 custodial grandmothers , facilitated by a doctoral-level community nutritionist. The major themes summarized from the qualitative group interviews were framed within a feminist/race theoretical context. Dominant themes from the focus group encounters include traditional gender roles related to food purchase and preparation, prioritizing food options to meet family preferences, available/accessible urban-based food options, food knowledge deficits, and sustaining cultural identity and nutritional health. Findings suggest implications for food and health policy, community-level programming, and nutrition education interventions.

